# A Study of Lipid Profile and the Correlation of Serum Uric Acid Levels in Patients With Hypertension

**DOI:** 10.7759/cureus.62952

**Published:** 2024-06-23

**Authors:** Muskaan Ahlawat, Sachin Shivnitwar, Akshata Borle, Sai Priya Ande, Sandesh Raut

**Affiliations:** 1 Internal Medicine, Dr. D. Y. Patil Medical College, Hospital & Research Centre, Dr. D. Y. Patil Vidyapeeth (Deemed to be University), Pune, IND

**Keywords:** elevated uric acid levels, high blood pressure, lipid profiles, hypertension in the global context, serum uric acid level

## Abstract

Aim

We examine the lipid profile and correlation of serum uric acid (SUA) levels in cases of hypertension and normotensives.

Methods

The current observational study spanned between April 2022 and April 2024. Throughout the research, 200 patients were examined; 100 of these patients were classified as Stage 1 or Stage 2 hypertensive (as per the seventh report of the Joint National Committee on Prevention, Detection, Evaluation, and Treatment of High Blood Pressure), while the other 100 served as controls, meaning they did not have hypertension or any other medical condition that could lead to elevated SUA levels.

Results

It was revealed that the proportion of hypertension was higher in males compared to females. Of the total male patients, most (41.1%) patients had grade 1 hypertension and grade 2 hypertension, while among females, 20% had grade 1 hypertension. It was seen that as age increases, systolic blood pressure (SBP) and diastolic blood pressure (DBP) also rise among the two study groups, although the correlation was not statistically significant between blood pressure level and age of study subjects. The hypertensive patients have increased SBP and DBP levels when compared to the control group, which is significant. The lipid profile shows that the hypertensive subjects had significantly higher mean low-density lipoprotein (LDL), very low-density lipoprotein (VLDL), and triglyceride levels than controls. SUA levels were observed to be elevated in the hypertensive subjects implying a positive correlation between the level of uric acid and blood pressures.

Conclusion

We found evidence that hyperuricemia and hypertension go hand in hand. A statistically noteworthy positive connection was found between the systolic blood pressures and lipid profiles of the patients. Hypertensive patients were found to have hyperlipidemia, whereas normotensive controls had normal lipid profiles. Moreover, it was seen that there was a positive correlation between SBP and chronological age in hypertensive cases, although this was statistically not significant.

## Introduction

Because of its prevalence and the dangers of developing other cardiovascular and chronic renal diseases, hypertension is a major global public health problem [1]. Research has linked increased serum uric acid (SUA) levels to an increased risk of developing metabolic syndrome, hypertension, diabetes, obesity, renal failure, and diabetes [2-4]. Many people with hypertension do not get proper treatment because they do not comprehend the need to lower their blood pressure to reduce the risk of systemic complications [5,6]. People who have high blood pressure often end up with kidney disease, among other complications [7]

Several health issues, including cardiovascular death, central nervous system ischemia, congestive cardiac failure, renal failure, and a log-linear relationship between blood pressure and these conditions were demonstrated by the Asia Pacific cohort studies collaboration. This relationship remains at least down to 115/75 mmHg [8]. As part of the metabolic syndrome, hypertension is associated with decreased high-density lipoprotein (HDL) levels and increased triglyceride, cholesterol, low-density lipoprotein LDL, and VLDL levels [9]. Endogenous purine molecules account for 2/3 of blood uric acid, whereas dietary sources account for 1/3. Uric acid is a metabolic by-product of purine metabolism. Both the presence and concentration of uric acid are known to contribute to the development of hypertension, and uric acid is considered a separate risk factor for hypertension [10-12]. Inflammation, endothelial dysfunction, the proliferation of vascular smooth muscle cells in renal microcirculation, and activation of the renin-angiotensin-aldosterone system (RAAS) are some of the pathogenic activities linked to uric acid levels between 10 and 12 that cause hypertension [6].

In 1957, a household with a unique lineage visited Hammer Smith Hospital, and the doctors there discovered a link between hypertension and hyperuricemia. Among the father's seven siblings, six of them had hyperuricemia, while the mother's whole family suffered from hypertension [13]. Because of its impact on serious cardiovascular events, its association with other risk factors, and its high incidence (about 40% of people), hypertension is a significant risk factor for heart disease [14]. Mahomed postulated in the 1800s that elevated SUA might impact the control of blood pressure [15]. More and more studies have come out since this earlier discovery, suggesting that people are coming to a more unanimous conclusion on the close connection between uric acid and hypertension [16].

The present study set out to determine if hypertensive individuals had higher uric acid levels and, if so, how this relates to their blood pressure. The motivation behind doing this study was to identify methods that can be implicated in lifestyle changes to avoid hypertension and its sequelae.

## Materials and methods

The observational research was conducted from April 2022 to April 2024. During the study, 200 patients were examined. Among them, 100 patients had Stage 1 or Stage 2 hypertension, and the other 100 were controls without hypertension or any other condition that could raise SUA levels.

Participants in the study included all consecutive hypertensive patients who visited the medical outpatient clinic, were admitted to the medical wards, or were referred from other outpatient departments.

Individuals who passed the inclusion criteria and did not come under any of the exclusion criteria were included.

Data were collected from one hundred patients who were categorized as cases. These examples include hypertension patients who are currently on antihypertensive medication and newly diagnosed hypertensive patients who have never received treatment and visited general outpatient clinics. A hundred healthy persons of similar age and sex were randomly chosen from a hospital and categorized as controls.

Inclusion criteria 

The inclusion criteria of the study are presented in Table 1.

**Table 1:** Inclusion criteria of the study

Inclusion criteria
Without regard to whether they were undergoing therapy or not, the study encompassed all persons with a hypertension diagnosis
Patients above the age of 30 years
A patient who gives their verbal or written consent after being informed
Individuals who do not exhibit any symptoms of cardiovascular disease, serious valve problems, secondary hypertension, kidney failure, diabetes mellitus, or any other systemic illness.
Patients who are not currently taking medication to decrease their cholesterol
People who are not taking medication to decrease their uric acid levels

Exclusion criteria 

The exclusion criteria of the study are presented in Table 2.

**Table 2:** Exclusion criteria of the study

Exclusion criteria
People who are taking medicine to decrease their cholesterol levels.
Patients who have diabetes
Individuals showing symptoms of secondary hypertension in either the clinic or the lab
Patients who are younger than 30 years old
Patients presenting with the clinical problems listed above
Women who are pregnant or using oral contraceptives
People whose medication regimen includes a decrease in uric acid
Nonarticular symptoms of hyperuricemia, such as gout and others
History of thiazide diuretics and other medications with the potential to induce hyperuricemia

Data collection

All subjects of cases (Group A) and controls (Group B) were medically examined as per a fixed format. Physical examination included the following measurements.

Height

Using a vertical height board that had a metric scale, the height was determined. Each foot should be evenly weighted, with the heels touching, and the individual should stand barefoot on a level surface with their head angled such that their vision is perpendicular to the body. It was made sure that each foot made contact with the upright board. The hair was flattened by pressing the headboard against the skull, and the measurement was obtained at the nearest 0.1 cm.

Weight

The patient was asked to stand on a dial type of weighing machine with their weight evenly distributed to register their weight.

BMI

It was calculated using the formula = weight in kg/(height in meters).

Blood Pressure

A sphygmomanometer is the tool that was utilized. An electrocardiogram (ECG), chest X-ray, fasting lipid profile, and urine analysis for routine examination were among the many tests that were part of the clinical investigation that aimed to ascertain the patient's health status. Fasting SUA levels were also investigated.

No smoking and no vigorous exercise for at least two hours were to be observed by the patients in the twelve hours preceding their examinations. On three separate occasions, the patient's systolic and diastolic blood pressures were taken through the right arm at the level of the heart in a sitting position using a standard sphygmomanometer. Following a brief period of relaxation in a peaceful setting, this was executed. When taking the anthropometric measurements, the subject was dressed in loose-fitting hospital clothing. Their height and weight were among the taken.

Statistical analysis

The data was analyzed with an Excel spreadsheet and with the help of Statistical Product and Service Solutions (SPSS, version 20.0; IBM SPSS Statistics for Windows, Armonk, NY).

## Results

It was revealed that the proportion of hypertension was higher in males compared to females. There were 56% males and 44% females among the hypertensive patients, indicating an increased predisposition of males toward hypertension especially after 60 years. The highest proportion of hypertension (39.3%) was found in the geriatric age group (i.e., more than 60 years). The mean age of cases with hypertension was 52.6±11.8 years. Among normotensive controls, the male participation was 52%, and the female participation was 48%. The mean age of study subjects without hypertension was 51.7±10.6 years (Table 3).

**Table 3:** Age and sex-wise distribution in study subjects SD: standard deviation

Age group (years)	Hypertensive cases		Total	Normotensive control		Total
	Male (%)	Female (%)		Male (%)	Female (%)	
30-39	08 (14.3)	09 (20.5)	17 (17)	03 (5.8)	10 (20.8)	13 (13)
40-49	11 (19.5)	11 (25.0)	21 (21)	14 (26.9)	17 (35.4)	31 (31)
50-59	15 (26.8)	09 (20.5)	28 (21)	17 (32.7)	10 (20.8)	27 (27)
≥60	22 (39.3)	15 (34.1)	38 (38)	18 (34.6)	11 (22.9)	29 (29)
Total	56 (100)	44 (100)	100 (100)	52 (100)	48 (100)	100 (100)
Mean ± SD	53.4±11.7	51.7±11.9		54.7±10.2	48.1±10.1	
Mean age	52.6±11.8		-	51.7±10.6		-

The mean value of SUA among the control group was 4.71±1.31, whereas, in some cases, it was 7.50±1.42. This shows that hypertensive patients have increased SUA levels when compared to the control group, which is statistically noteworthy (p value <0.05). This depicts the prevalence of elevated SUA with hypertension. In the control population, normal uric acid levels are presented in Table 4.

**Table 4:** Comparison of SUA levels among hypertensive cases and normotensive controls SD: standard deviation; SUA: serum uric acid

Variable	Hypertensive (Mean±SD)	Normotensive (Mean±SD)	t-statistic	Statistical significance (P value)
SUA	7.50±1.42	4.71±1.31	14.366	0.000
Minimum-maximum	3.0-9.8	2.0-7.2		

The mean value of LDL among the control group was 91.60±21.44, whereas, in other cases, it was 140.33±48.52. The lipid profile shows that the hypertensive subjects had significantly higher mean LDL, VLDL, and triglyceride levels than controls. The mean HDL was lower in the hypertensive than control groups (36.74±11.26 versus 51.40±9.12, t=−10.22, p value <0.05) (Table 5).

**Table 5:** Comparison of fasting lipid profile between controls and cases LDL: low-density lipoprotein; HDL: high-density lipoprotein; VLDL: very low-density lipoprotein; SD: standard deviation

Variable	Hypertensive (Mean±SD)	Normotensive (Mean±SD)	t-statistic	Statistical significance (P value)
LDL level	140.33±48.52	91.60±21.44	15.729	0.000
VLDL level	41.11±9.10	25.74±7.42	16.377	0.000
HDL level	36.74±11.26	51.40±9.12	-10.226	0.000
Triglyceride	218.17±42.52	127.26±37.83	15.972	0.000

It was seen that, as age increases, SBP and DBP also increase among both study groups, although the correlation was not statistically significant between blood pressure level and age of the study subjects (Table 6).

**Table 6:** Age-wise mean blood pressure of cases and controls

Age group of cases	Frequency	Mean SBP	Mean DBP
30-39	17	154.24±20.89	98.94±8.18
40-49	22	155.64±13.23	100.91±10.39
50-59	24	161.33±13.81	98.83±9.90
≥60	37	162.05±15.55	100.27±6.89
Total	100	159.14±15.84	99.84±8.63
Age group of controls			
30-39	13	113.08±7.69	75.69±6.10
40-49	31	114.26±5.05	77.91±6.18
50-59	27	115.04±06.47	79.56±5.52
≥60	29	116.90±4.82	81.17±4.58
Total	100	115.08±5.83	78.98±5.78

Among hypertensive patients, there was a significant correlation between SUA and LDL (r=0.269, p=0.007) and triglyceride (TG) (r=0.223, p=0.025). No significant correlation was found in any of the lipid parameters in normotensive patients (Table 7).

**Table 7:** Correlation between serum fasting lipid profile and SUA levels LDL: Low-density lipoprotein; HDL: High-density lipoprotein; VLDL: Very low-density lipoprotein; SUA: Serum uric acid P value was obtained through the following statistical test: ANOVA.

Group	Lipid profile	Correlation (r)	P value
Hypertensive cases	LDL	0.269	0.007
	VLDL	0.084	0.405
	HDL	0.071	0.447
	Triglyceride	0.223	0.025
Normotensive control	LDL	0.161	0.110
	VLDL	0.077	0.448
	HDL	0.115	0.254
	Triglyceride	-0.101	0.319

## Discussion

Hyperuricemia is commonly linked to lifestyle-related illnesses [17,18]. Between 25% and 40% of untreated hypertensive patients also have high levels of uric acid in their blood [19,20]. Several major epidemiological studies have shown a connection between high SUA levels and hypertension in adults [21,22].

The average age of our study participants was higher than the average age reported in the studies by Eisen et al. and Grayson et al. [23,24]. The gender ratio (male/female) in our study was 1.12:1, which was lower than the ratio of 1.5:1 reported by Feig et al. [25]. The mean SBP and DBP in our patients were 137.11±25.09 and 89.41±12.76, respectively. These values were lower than those reported in the study by Feig et al., where the mean SBP and DBP were 139 mm of mercury and 83 mm of mercury, respectively. As individuals age, there is a noticeable increase in blood pressure. Individuals above the age of 60 exhibit a higher increase in blood pressure levels. A higher prevalence of individuals with hypertension was noted in the 50-59 age range.

Elevated SUA has been linked to a higher likelihood of developing cardiovascular disease. SUA can directly impair cardiovascular health by enhanced platelet assemblage and inflammatory stimulation of the endothelium [26]. There is a controversy about whether increased blood uric acid level alone increases cardiovascular risks because it correlates with many risk factors such as hypertension, renal dysfunction, insulin resistance, hyperlipidemia, and hyperhomocysteinemia. Additional reports have demonstrated elevated SUA levels in individuals with hypertension. A study found that 46% of the 400 hypertension patients in their study population had hyperuricemia [27]. Ample data indicate that uric acid is more sensitive and serves as an earlier indication for renal failure compared to creatinine. SUA plays a part in the production of free radicals. Free radicals impede the expansion of blood vessels in the endothelium. Antioxidant medicines demonstrate a blood pressure-reducing impact. Multiple observations corroborate the scheme of free radical-induced suppression of endothelium-dependent vasodilation. Inadequate intake of antioxidants in the diet can lead to hyperuricemia, and it is linked to the development of hypertension. Antioxidant medications have been found to decrease blood pressure in individuals with diabetes and hypertension [28]. Tykarski found that SUA levels and the frequency of hyperuricemia were notably greater in hypertensive individuals. The study showed that the tubular secretion of UA was markedly reduced in hypertensive individuals compared to those with normal blood pressure. There was no variation in the reabsorption of uric acid before and after secretion. The significant frequency of hyperuricemia in hypertension was attributed to poor renal secretion of UA, according to their conclusion [29]. Goldstein et al. showed that, in an adolescent population, SUA notably predicted blood pressure even after controlling for age, weight, height, and sexual maturity [30].

This study did not find a significant correlation between SUA and BMI. Shobakaelker et al. and Healiey showed in their research that there was no notable association between obesity and SUA level. While uric acid possesses antioxidant qualities, it exhibits high oxidant effects in the presence of obesity. Oxidative stress from elevated SUA levels and inflammation in obesity can both contribute to an increased risk of hypertension in patients. Metabolic syndrome has also been shown to significantly increase the risk of hypertension.

We discovered a clear correlation in SUA levels in hypertensive subjects. Therefore, the potential role of SUA in generating free radicals and inducing oxidative stress, resulting in renal dysfunction because of nephrosclerosis and elevated levels of SUA, should be considered. The limitations of the study are presented in Table 8.

**Table 8:** Limitations of the study

Limitations of the study
Cross-sectional design. Our study was a cross-sectional study, which is not sufficient to confirm the relationship between SUA and hypertension. A longitudinal study is needed to confirm the same.
Single-center study. Conducting the study at a single center limits the diversity of the sample, which may not capture variations across different regions.
Confounding factors. Although efforts were made to control for various factors, other potential confounding variables (genetic predisposition, environmental factors, and so on might not have been fully accounted for, impacting the outcomes.
Measurement limitations. The study used standard clinical methods for measuring blood pressure. Variations in these measurements could arise because of technical factors or individual physiological differences.

Several studies have also demonstrated a positive correlation between SUA levels and hypertension. Controversies exist on whether elevated uric acid is an independent risk factor for heart disease. It is recommended to conduct prospective studies on newly diagnosed hypertensive individuals to determine the validity of this claim.

## Conclusions

This study implies that dietary habits play a vital role in predicting hypertension among the population. A diet low in SUA (i.e., less red meat and alcohol) can significantly reduce the burden of hypertension and cardiovascular complications at the global level. A statistically noteworthy positive connection was found between systolic blood pressure and the lipid profiles of the patients. Lowering lipid levels in individuals should be aimed at controlling hypertension and preventing further coronary artery diseases among individuals. Lifestyle modifications should be stressed upon the patients presenting with elevated blood pressure to prevent hypertension and its consequences. We determined that there is a direct correlation between hyperuricemia and hypertension. It was observed that there was a positive link between systolic blood pressure (SBP) and chronological age in hypertensive individuals.
